# Effects of Transcutaneous Electrical Acupoint Stimulation on the Incidence of Hypoxia in Elderly Patients Undergoing Painless Gastrointestinal Endoscopy: A Randomized Controlled Trial

**DOI:** 10.1155/prm/1251246

**Published:** 2024-12-22

**Authors:** Wenyu Zhou, Yu Wang, Pengcheng Ye, Song Hu, Siyu Li, Mingxia Wang, Duanyang Sheng, Yuanli Chen, Wang Shen, Yi Zhang, Feng Liu, Wei Zhang, Xin Lv, Xiangrui Wang, Hao Yang

**Affiliations:** ^1^Department of Anesthesiology, Shanghai Pulmonary Hospital, Tongji University School of Medicine, Shanghai 200433, China; ^2^Department of Anesthesiology and Pain Management, Shanghai East Hospital, Tongji University School of Medicine, Shanghai 200120, China; ^3^Department of Anesthesiology, Tongren Hospital, Shanghai Jiao Tong University School of Medicine, Shanghai, China

**Keywords:** elderly patients, hypoxia, painless gastrointestinal endoscopy, transcutaneous electrical acupoint stimulation

## Abstract

**Background:** Hypoxia is not uncommon in elderly patients during painless gastrointestinal endoscopy. This study aimed to determine the effectiveness of transcutaneous electrical acupoint stimulation (TEAS) in reducing the occurrence of hypoxia symptoms in elderly patients.

**Methods:** Patients were randomly and equally grouped into sham control (*n* = 109) or TEAS group (*n* = 109) by using the random number table method. Patients in the TEAS group received electrical stimulation at the bilateral ST36 points 30 min before the examination until the end of the painless gastrointestinal endoscopy. Patients in the control group only had electrodes attached to bilateral nonacupoints in a similar pattern as the TEAS group without electrical stimulation. The primary endpoints measured were the incidence of hypoxia and severe hypoxia. The secondary endpoints included propofol dosage, sedation-related adverse events, hemodynamic parameters, surgical duration, patient recovery time, pain score, patient satisfaction, anesthesiologist satisfaction, and endoscopist satisfaction.

**Results:** Of the 251 patients who participated in this study, 218 patients ended up completing the final study. The primary outcome was that, compared with group control, the incidence of hypoxia in group TEAS was reduced by 11% (19.3% vs. 8.3%, *p*=0.018) and the incidence of severe hypoxia did not show a significant change (7.3% vs. 2.8%, *p*=0.122). And there was a significant decrease in the occurrence of patients requiring emergency airway assistance (increased oxygen flow: 16.5% vs. 6.4%, *p*=0.019, jaw thrust: 11.0% vs. 3.7%, *p*=0.038, mask-assisted ventilation: 5.5% vs. 1.8%, *p*=0.015).

**Conclusion:** TEAS can reduce the incidence of hypoxia in elderly patients undergoing painless gastrointestinal endoscopy.

**Trial Registration:** ClinicalTrials.gov identifier: ChiCTR2200059465.

## 1. Introduction

Gastrointestinal endoscopy is an important method for diagnosing digestive tract diseases and plays a vital role in preventing serious conditions such as gastric cancer and colorectal cancer [1]. In line with the promotion of comfortable medical care, painless gastrointestinal endoscopy is gradually replacing the traditional approach. Among the various sedatives used, propofol is widely recommended due to its rapid onset, short half-life, and effective sedative effect in painless gastrointestinal endoscopy [2].

However, the use of propofol can lead to adverse events such as respiratory depression, hypoxia, hypotension, and arrhythmia, especially in elderly patients [3]. One of the most significant complications observed during painless gastrointestinal endoscopy with propofol-assisted sedation is hypoxia (where pulse oxygen saturation (SpO_2_) is between 75% and < 90% for less than 60 s), with an incidence rate as high as 44%–70% [4–6]. Although one study has shown that supraglottic oxygenation and ventilation can reduce the occurrence of hypoxia during painless gastrointestinal endoscopy with propofol sedation, this approach is challenging for older adults and may lead to complications in the upper respiratory tract or dental damage [7].

The application of acupuncture and moxibustion in China has a long history dating back 2000 years [8]. In recent years, transcutaneous electrical acupoint stimulation (TEAS) has gained attention in global clinical practice. TEAS is a noninvasive method that uses the electrical stimulation of acupuncture points through surface electrodes. It is simpler and safer compared to traditional electrical stimulation methods [9–11]. Studies have shown that perioperative TEAS administration can promote early postoperative recovery in elderly patients by reducing inflammation and stress response [12]. It has also been found to be effective in reducing abdominal pain after colonoscopy and relieving rectal discomfort caused by gas inflation. Applying TEAS at specific frequencies and acupuncture points may even help reduce the rate of hypoxia during colonoscopy in elderly patients [13–15].

In this research, we conducted a prospective, single-center, double-blind randomized controlled trial to evaluate the effect of TEAS on older adults undergoing painless gastrointestinal endoscopy. This study compared patients who received TEAS at ST36 (Zusanli) with those who did not receive electrical stimulation. Our primary objectives were to assess the incidence of hypoxia and complications and to provide experimental evidence for the clinical use of TEAS in this study.

## 2. Method

### 2.1. Study Design

This study is a prospective, single-center, patient- and investigator-blinded, randomized trial enrolling elderly patients who underwent painless gastrointestinal endoscopy at the Gastrointestinal Endoscopy Center of Shanghai East Hospital, Tongji University. The study period was from April 2022 to June 2023. Ethical approval for the study protocol was obtained from the Ethics Committee of Shanghai East Hospital [No. 2018 (589)]. Participants were selected preoperatively and were required to sign an informed consent form prior to enrollment in the study. This study adhered to the guidelines for reporting parallel group randomized trials, the Consolidated Standards for Reporting Trials 2010 (CONSORT) [16].

### 2.2. Participants

Eligible elderly patients undergoing painless gastrointestinal endoscopy were evaluated for inclusion between April 2022 and June 2023. Inclusion criteria were as follows: (i) age ≥ 65 years; (ii) patients undergoing painless gastrointestinal examination; and (iii) American Society of Aneshesiologists (ASA) score I–II. Exclusion criteria were as follows: (i) severe cardiac, pulmonary, hepatic, or renal dysfunction; (ii) cardiac arrhythmias with second- or third-degree atrioventricular block; (iii) chronic obstructive pulmonary disease and recent asthma attacks, or with hypoxia (pulse oxygen saturation (SpO_2_) < 90%); (iv) unsatisfactory control of hypertension (systolic blood pressure (SBP) > 160 mmHg or diastolic blood pressure (DBP) > 100 mmHg), or hypotension (SBP < 90 mmHg); (v) having a central nervous system disorder or neuropsychiatric disorder, such as epilepsy; (vi) using sedatives, sleeping pills or pain medication for more than 3 months; (vii) bradycardia (heart rate (HR) < 50 bpm/min) or tachycardia (HR > 100 bpm/min); (viii) allergy to propofol; and (ix) refusal to sign an informed consent form.

### 2.3. Study Protocol

#### 2.3.1. Randomization

Randomized grouping is done by the random number table method. Briefly, first we labeled all patients sequentially. Then, the starting point of sampling and the order of sampling are arbitrarily specified on the random number table. At last, sample numbers are drawn sequentially from the random number table. The first 109 numbers are for the control group, and the next 109 numbers are for the TEAS group. The patient grouping was known only to the nurse anesthetist and research assistant; neither the patient nor the anesthesiologist nor the gastroscopist was aware of the patient grouping. The Hwato electrical stimulator [Specification: SDZ-III, Suzhou Medical Limited Company, Jiangsu Food and Drug Administration (Approval) No. 2270675, 2017] was placed in an opaque box in advance. And the anesthesiologists were unaware whether electrical stimulation was administered to the patient or not. Electrical stimulation was administered by anesthesia nurses who were not involved in patient care or study variables.

#### 2.3.2. Preoperative Preparation

All patients routinely fasted and abstained from food and drink for 8 h before painless gastrointestinal endoscopy, and the peripheral vein of the right upper limb was opened in the Gastrointestinal Endoscopy Center with a slow drip of 50 mL of sodium lactate Ringer's solution. The patient was placed in the left lateral decubitus position on the examination bed and inhaled oxygen (2 L/min) through a nasal cannula. The HR, blood pressure, electrocardiogram (ECG), and pulse oximetry were detected and recorded after the patient rested for 5 min.

#### 2.3.3. Trial Process

Patients in the TEAS group received high-frequency sparse-wave electrical stimulation at the bilateral ST36 points 30 min before the examination, with alternating pulses of 2 Hz and 100 Hz and a stimulation intensity of 15–25 mA until the end of the gastrointestinal endoscopy. Patients in the control group only had electrodes attached to bilateral nonacupoints in a similar pattern as the TEAS group without electrical stimulation. The bilateral nonacupoint locations were located in the vicinity of the bilateral Zusanli acupoints. This was confirmed by a professional and experienced acupuncturist. Patients cannot separate the difference between these two positions.

#### 2.3.4. Anesthesia and Procedures

The anesthesiologist induced anesthesia by slow peripheral intravenous (IV) injection of sufentanil (5 *μ*g) and propofol (2 mg/kg) to the patient and continuously observed the patient's state of consciousness. The Modified Observer Alertness/Sedation Assessment Scale (MOAA/S) was used to assess the depth of sedation [17]. Gastrointestinal endoscopy was initiated when the patient lost consciousness and had a MOAA/S score ≤ 2. Propofol 20–30 mg was given intravenously to maintain depth of sedation when the patient had a MOAA/S score ≥ 3 during the examination or when characteristic facial pain, limb movements, and hemodynamic changes were present. During sedation, all patients breathed spontaneously with 2 L/min of oxygen via nasal cannula. If the patient develops decreased oxygen saturation and the anesthesiologist rules out interference from the volumetric pulse waveform, the nasal cannula oxygen flow is increased from 2 to 6 L/min, while the mandible is lifted bilaterally until SpO_2_ is ≥ 95%, using mask ventilation to assist with breathing if necessary and tracheal intubation if severe hypoxia cannot be relieved. Phenylephrine (40 *μ*g, IV) was given if hypotension (SBP < 90 mmHg or 20% decrease in basal values) persisted for ≥ 1 min. If bradycardia (HR < 50 bpm/min) is present, give atropine (0.5 mg, IV). The procedure was repeated if needed. After the procedure, the patient was taken to the postanesthesia care unit (PACU) for further observation.

The HR, SBP, DBP, ECG, SpO_2_, and respiratory rate (RR) of the subjects were recorded at 30 min before anesthesia (at the time of receiving electrical stimulation), at the beginning of the examination (at 30 min of electrical stimulation), during intraoperative hypoxia, and at the time of consciousness recovery, respectively. When subjects had SpO_2_ ≤ 95% and ≤ 90% intraoperatively and during recovery of consciousness, the number of occurrences, their duration, and the corresponding interventions were recorded. The time of gastrointestinal endoscopy (time from the entry of the gastroscope into the mouth to the end of the examination, and the time from the entry of the endoscope into the anus to the end of the examination), the time of consciousness recovery (time from the end of the examination to the consciousness recovery), and the number of occurrences of postoperative nausea and vomiting were recorded for the subjects. The visual analog scale (VAS) was used to assess the level of postoperative pain and fatigue, patient, anesthetist, and endoscopist satisfaction.

### 2.4. Measurements

The primary endpoints measured were the incidence of hypoxia (SpO_2_ ≤ 95% and ≤ 90%) and severe hypoxia (SpO_2_ < 75% or 75% ≤ SpO_2_ < 90% for > 60 s). The secondary endpoints were: propofol consumption (induction dose, maintenance dose, and total dose), the flow of oxygen (increase the flow of oxygen to 6L min-1), intraoperative hemodynamic parameters (SBP, DBP, mean arterial pressure (MAP), HR), and RR, the time of gastrointestinal endoscopy, the time of consciousness recovery, occurrences of sedation-related adverse events (including hypotension, bradycardia, emergence agitation, cough, hiccups, nausea, and vomiting), postoperative pain score, postoperative fatigue, and satisfaction of patient, anesthetist and endoscopist. The personnel involved in the evaluation of these variables were not aware of the patient subgroups.

### 2.5. Sample Size

The sample size was calculated using Pass software, Version 15.0 (NCSS, Kaysville, UT, USA). Based on the results of a pilot study, the incidence of hypoxia during gastrointestinal endoscopy was 19% in the control group and 6% in the TEAS group [7]. Assuming a two-sided *α* = 0.05 and statistical power of 0.8, the sample size was calculated to 99 patients in each group. Considering a loss to follow-up of 10%, 109 patients were required for each group.

### 2.6. Statistics

Statistical analyses were performed after excluding patients who were considered ineligible after enrollment. All data were checked for normal distribution using the Shapiro–Wilk test. Normally distributed data were expressed as mean ± standard deviation, and non-normally distributed data were expressed as median with interquartile range. Continuous variables were analyzed using Student's *t*-test or Mann–Whitney *U* test. Categorical variables were compared using Pearson's *χ*^2^ test or Fisher's exact test, as appropriate. *p* value less than 0.05 was considered statistically significant. Data analyses were conducted using SPSS 24.0 (SPSS Inc., Chicago, IL, USA).

## 3. Results

**Figure 1:**
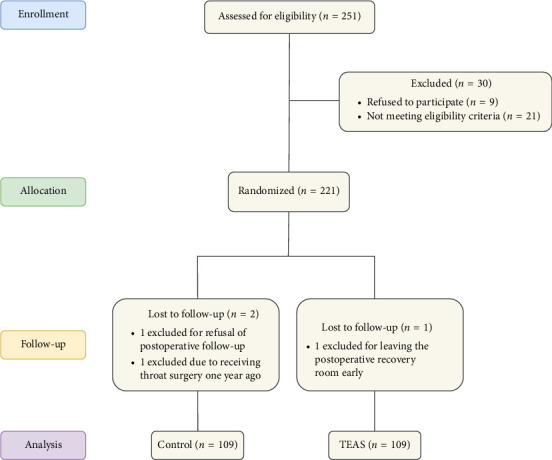
CONSORT flowchart of the procedures for patients undergoing gastroscopy.

During the study period from April 2022 to June 2023, a total of 251 patients were assessed for inclusion in this study. Among them, 30 patients were excluded, including nine who declined to participate and 21 who did not meet the eligibility criteria. Ultimately, 221 patients were randomly assigned to two groups and successfully completed gastrointestinal endoscopy. However, three patients were later excluded in the final analysis. The reasons for exclusion were as follows: one patient refused postoperative follow-up, one patient had undergone pharyngeal surgery a year earlier, and another patient left the postoperative recovery room early. Therefore, the final analysis included 218 patients (Figure 1). Both groups exhibited similar baseline characteristics, as shown in Table 1.

## 4. Primary Outcome

In comparison with the control group, the group receiving TEAS showed a notable 11% reduction in the incidence of hypoxia (19.3% vs. 8.3%, *p*=0.018, Table 2). Additionally, the incidence of severe hypoxia decreased, but there was no statistically significant difference (7.3% vs. 2.8%, *p*=0.122, Table 2). Moreover, there was a significant decrease in the occurrence of patients requiring emergency airway assistance, such as increased oxygen flow (16.5% vs. 6.4%, *p*=0.019, Table 2), jaw thrust (11.0% vs. 3.7%, *p*=0.038, Table 2), and mask-assisted ventilation (5.5% vs. 1.8%, *p*=0.015, Table 2). On the other hand, there was no significant difference observed in intubation rates.

### 4.1. Secondary Outcome

Group TEAS demonstrated a significantly lower propofol maintenance dose compared to group control (52.0 (42.0, 61.0) vs 45.0 (26.0, 57.5), 95%CI: 6(0, 12), *p*=0.011, Table 3). However, there were no significant differences in the total propofol dose and induction dose between group control and group TEAS.

Moreover, group TEAS exhibited a reduced patient procedure time (15.7 (13.1, 16.9) vs 12.5 (10.4, 14.5), 95% CI: 2.3(1.7, 3.2), *p* < 0.001, Table 3) and awakening time (4.5 (4.2, 5.2) vs 4.3 (3.3, 4.8), 95% CI: 0.5(0.25, 0.73), *p* < 0.001, Table 3) compared to group control.

**Figure 2:**
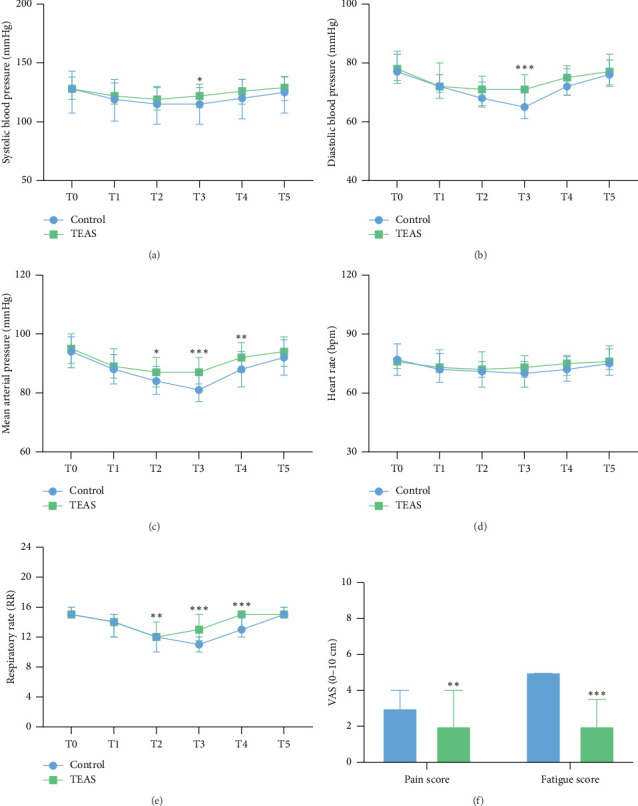
Hemodynamic parameters, respiratory rate, postoperative pain score, and fatigue score of patients. Notes: (a) systolic blood pressure; (b) diastolic blood pressure; (c) mean arterial pressure; (d) heart rate; (e) respiratory rate; (f) postoperative pain and fatigue scores. T0 = Before the induction of sedation; T1 = at the beginning of the procedure; T2 = at 3 min after the start of the procedure; T3 = at 6 min after the start of the procedure; T4 = at the end of the procedure; T5 = after the recovery of consciousness. ⁣^∗^*p* < 0.05 vs control, ⁣^∗∗^*p* < 0.01 vs control, ⁣^∗∗∗^*p* < 0.001 vs control.

Hemodynamic parameters showed minimal differences between the two groups. Only SBP and DBP at T3 and MAP at T2–T4 varied significantly (SBP T3: *p* < 0.05, DBP T3: *p* < 0.001, MAP T2: *p* < 0.05, T3: *p* < 0.001, T4: *p* < 0.01, Figures 2(a), 2(b), and 2(c)). Importantly, group TEAS demonstrated smoother hemodynamic parameters. The HR did not differ significantly, but group TEAS exhibited greater stability in the RR (T2: *p* < 0.01, T3: *p* < 0.001, T4: *p* < 0.001, Figures 2(d) and 2(e)).

Furthermore, pain scores and fatigue scores assessed by the VAS were significantly lower in group TEAS compared to group control (Figure 2(e)). There were no notable differences in sedation-related adverse events, such as hypotension, bradycardia, emergence agitation, cough, hiccups, nausea, and vomiting (Table 4). Patient satisfaction, anesthesiologist satisfaction, and endoscopist satisfaction were all higher in group TEAS than in group control (patient satisfaction score: *p* < 0.001, anesthetist satisfaction score: *p* < 0.001, endoscopist satisfaction score: *p* < 0.001, Table 5).

## 5. Discussion

This study demonstrated that performing TEAS at ST36 can lowered the incidence of hypoxia and emergency airway management in elderly patients undergoing painless gastrointestinal endoscopy. It also reduced the maintenance dose of propofol, decreased the duration of gastrointestinal endoscopy and patient recovery time, improved patient satisfaction, and alleviated postoperative pain. Additionally, the use of TEAS improved the satisfaction of anesthesiologists and gastroenterologists performing the endoscopy. Furthermore, there were no adverse effects associated with the TEAS treatment. Our results suggested that TEAS can be widely promoted for clinical application.

As the trend of population aging accelerates and people's demand for health increases, regular physical examinations have gradually become one of the main ways for elderly people to maintain their health. Among them, the demand for gastrointestinal endoscopy is also increasing. Painless gastrointestinal endoscopy has advantages such as painlessness, high accuracy, short examination time, and minimal trauma. By undergoing gastrointestinal endoscopy, elderly patients can detect suspicious lesions early or directly remove affected tissues. However, due to age-related decline in cardiopulmonary function and the presence of various underlying diseases, elderly patients are prone to experiencing serious complications during painless gastrointestinal endoscopy, such as hypoxia and cardiac arrhythmias. Therefore, it is crucial to explore new drugs or methods that can be used in combination with IV general anesthetic drugs, such as propofol, making the procedure safer for elderly patients undergoing gastrointestinal endoscopy.

Acupuncture can alleviate discomfort during colonoscopy examinations [18]. However, acupuncture is an invasive and delicate procedure that requires skilled traditional Chinese medicine practitioners [19]. TEAS utilizes specialized electrode patches placed on the specific human acupoints to achieve its effects. Compared to traditional acupuncture, TEAS offers noninvasiveness, ease of standardization, and low cost. It also reduces the risk of infections associated with the procedure [20]. Electric acupuncture at ST36 has been found to significantly reduce patient discomfort and minimize adverse events during gastric endoscopy examinations [21].

In recent years, concerns about hypoxemia caused by propofol sedation have been increasing. However, there is currently no consensus in clinical practice regarding this issue. The use of propofol alone often does not achieve satisfactory anesthesia effects and can lead to adverse reactions such as respiratory depression and hypotension. In nonelderly patients, the incidence of hypoxia when performing propofol-induced painless gastrointestinal endoscopy is approximately 9%–16% [7, 22, 23]. In elderly patients, the incidence rate of hypoxia during propofol-induced painless gastrointestinal endoscopy is approximately 27.1% [24]. And the most common complications that occur in elderly patients during gastrointestinal endoscopy are hypoxia and hypoxemia [25]. TEAS at PC6 (Neiguan) has been proven to improve blood oxygen saturation and reduce the incidence of hypoxia in elderly patients during colonoscopy examinations, thus preventing the occurrence of hypoxemia [15]. Consistent with this, in our study, performing TEAS at ST36 has been found to effectively reduce the incidence of hypoxia in elderly patients during gastrointestinal endoscopy examinations. Additionally, there was a significant decrease in the need for emergency airway management measures such as mask-assisted ventilation and endotracheal intubation. As these measures interrupt the process of endoscopy, reducing their necessity greatly improved the satisfaction of both anesthesiologists and endoscopists. In painless gastrointestinal endoscopy, elderly patients are more susceptible to hypoxia due to respiratory depression and decreased hypoxia tolerance. We hypothesized that there are three possible mechanisms by which TEAS can reduce the incidence of hypoxia. First, TEAS can activate the vagus nerve, which helps maintain respiratory stability and reduce the risk of respiratory depression [26]. Second, TEAS can improve respiratory stability by stimulating relevant acupoints (e.g., Neiguan) to improve respiratory ventilation [15]. Finally, TEAS can relieve pain and anxiety and reduce the use of sedative medications [27].

A placebo-controlled randomized trial revealed that there was no significant difference in the amount of propofol and Alfentanil used during colonoscopy between the electroacupuncture and placebo groups [27]. However, a meta-analysis found that TEAS could reduce the total amount of propofol and remifentanil used in adult surgical patients during general anesthesia [28]. The combination of electroacupuncture and propofol sedation in gastrointestinal endoscopy can reduce the amount of propofol used and speed up patient recovery [29], which is consistent with our findings that the maintenance dose of propofol was significantly reduced in the TEAS group, but the induction and total doses did not change. This may be because electroacupuncture can reduce the patient's reactivity to painful stimuli during anesthesia, making them more tolerant to harmful stimuli [30]. Compared with general anesthesia surgery, painless gastrointestinal endoscopy requires a shorter time, and the reduction in the maintenance dose of propofol is relatively small. Therefore, in our results, the total amount of propofol did not decrease. In addition, the surgery time and recovery time were significantly shortened, which was closely related to the reduction in the maintenance dose of propofol by TEAS. These results suggest that TEAS can accelerate the turnover of painless gastrointestinal endoscopy and promote patient recovery.

After propofol induction, SBP, DBP, MAP, HR, and RR decreased to a certain extent and returned to baseline levels in both groups after recovery. During the maintenance period of anesthesia, the TEAS group had significantly more stable hemodynamic parameters and less respiratory depression than the control group. Pan et al. found that TEAS-assisted treatment resulted in more stable hemodynamic parameters and accelerated recovery in patients undergoing laparoscopic myomectomy [31]. On one hand, TEAS can reduce the amount of intraoperative general anesthetic drugs, which reduces the risks associated with anesthetic drugs. On the other hand, TEAS also has a good analgesic effect [32], which decreases the impact on the patient of the pain caused by the endoscopy operation. Therefore, the combination of TEAS and propofol-induced painless gastrointestinal endoscopy can make the hemodynamic parameters of elderly patients more stable, which can help prevent cardiovascular accidents in elderly patients with underlying diseases such as hypertension.

A recent meta-analysis showed that TEAS reduced the incidence of postoperative nausea and vomiting and reduced the number of patients requiring antiemetic resuscitation [33] and reduced the incidence of nausea and vomiting after laparoscopic nongastrointestinal surgery in patients undergoing high-risk procedures [34]. Our results show that TEAS did not significantly reduce the incidence of sedation-related adverse events. However, from the data in the table, it appears that the TEAS group experienced somewhat fewer sedation-related adverse events.

We used the VAS to assess the level of postoperative pain and fatigue, patient, anesthetist, and endoscopist satisfaction. Our results show that patients in the TEAS group had lower pain scores and fatigue scores compared with the control group. Agrawal et al. also found that TEAS reduced the use of opiates in patients with inguinal hernias and provided relief from postoperative pain [35]. A previous study also showed that receiving TEAS in the perioperative period relieved postoperative pain and improved patient satisfaction after laparoscopic surgery [36]. This is consistent with our results that TEAS improved patient satisfaction with gastrointestinal endoscopy. This may be due to the fact that TEAS alleviates patient pain and reduces the incidence of adverse events. In addition, we also found that the satisfaction of anesthetists and endoscopists was higher in the TEAS group, possibly because TEAS made the patient smoother during the operation and the doctor had fewer emergencies to deal with.

As patients and doctors become more familiar with TEAS, it is being used in more and more clinical scenarios. Wu et al. found that TEAS could alleviate preoperative anxiety states and reduce postoperative pain in patients undergoing thoracoscopic surgery [37]. Zhang et al. found that in patients undergoing laparoscopic cholecystectomy, TEAS accelerated the recovery of spontaneous urination and reduced the incidence of postoperative urinary retention [38]. The results of a multicenter randomized clinical study also showed that TEAS contributed to a reduction in the incidence of postoperative bowel paralysis and facilitated recovery of gastrointestinal function after rectal cancer surgery [39]. These results are consistent with our finding that patients can benefit from TEAS, resulting in a reduction in the incidence of adverse effects and accelerated recovery. Currently, researchers are focusing more on the study of TEAS in pain relief, gastrointestinal function recovery, and cognitive function. The exploration of hypoxia has not received special attention. Elderly patients undergoing gastrointestinal endoscopy have a higher incidence of hypoxia and are more prone to serious adverse events, so it is important to find ways to effectively reduce the incidence of hypoxia during the procedure.

There are still some limitations in this study. First, our study was not a multicenter study and the sample size was not large enough. This has the risk of affecting the reliability of the results of the statistical analysis and has the danger of increasing the risk of type one error. Second, in this study we mainly included relatively healthy (ASA I–II) elderly patients. Therefore, whether TEAS has a similar treatment effect in these patients who were at higher risk (ASA III) or who had recent acute high-risk disease needs to be further explored. Finally, we performed the TEAS procedure on patients 30 min before anesthesia, the patient can aware of the presence of TEAS, which may lead to a placebo effect in the TEAS group. To improve the reliability of the results of this study, we chose one researcher to record all the data, another to complete the data entry and statistical analysis, and both to be unaware of the specific groupings.

## 6. Conclusion

Our findings suggested that the use of TEAS during painless gastrointestinal endoscopy reduces the incidence of hypoxia in elderly patients, decreases the amount of propofol, and accelerates the recovery of patients. TEAS is a noninvasive, easy to perform, safe and reliable method that is worthy of further clinical application.

## Figures and Tables

**Table 1:** General characteristics of patients.

Characteristics	Control	TEAS	*p* value
Age [yr; mean (SD)]	70.97 ± 4.89	71.33 ± 4.38	0.570
Sex			0.892
Male [*n* (%)]	56 (51.4)	55 (50.5)	
Female [*n* (%)]	53 (48.6)	54 (49.50)	
BMI [kg m^−2^; mean (SD)]	23.57 ± 2.94	23.24 ± 2.66	0.388
ASA physical status [*n* (%)]			0.581
I	46 (42.2)	42 (38.5)	
II	63 (57.8)	67 (61.5)	
History of hypertension [*n* (%)]	38 (34.9)	35 (32.1)	0.667
History of diabetes [*n* (%)]	21 (19.3)	23 (21.1)	0.736
Mallampati score [*n* (%)]			0.527
I	41 (37.6)	43 (39.4)	
II	46 (42.2)	49 (45.0)	
III	19 (17.4)	12 (11.0)	
IV	3 (2.8)	5 (4.6)	
Mouth opening [*n* (%)]			0.832
I	0	0	
II	12 (11.0)	13 (11.9)	
III	97 (89.0)	96 (88.1)	
Basal SpO_2_ [%; mean (SD)]	98 ± 8.58	98 ± 1.19	0.256

*Note:* There were no significant differences between the two groups (*p* > 0.05). Mallampati score I/II/III/IV: I, faucial/tonsillar pillars, uvula, and soft palate are all visible; II, partial visibility of the faucial/tonsillar pillars, uvula, and soft palate; III, base of the uvula, and soft and hard palate visible; IV, only hard palate is visible; mouth opening I/II/III: I, one finger; II, two fingers; III, three fingers; SpO_2_, pulse oxygen saturation.

Abbreviations: ASA, American Society of Anesthesiologists; BMI, body mass index; TEAS, transcutaneous electrical acupuncture point stimulation.

**Table 2:** Respiratory-related adverse events and interventions.

Variables	Control	TEAS	*p* value
Hypoxia [*n* (%)]	21 (19.3)	9 (8.3)	**0.018**
Severe hypoxia [*n* (%)]	8 (7.3)	3 (2.8)	0.122
Increase the flow of oxygen [*n* (%)]	18 (16.5)	7 (6.4)	**0.019**
Jaw thrust [*n* (%)]	12 (11.0)	4 (3.7)	**0.038**
Mask-assisted ventilation [*n* (%)]	6 (5.5)	2 (1.8)	**0.015**
Intubation [*n* (%)]	0	0	0

*Note:* Statistically significant results are in boldface.

Abbreviation: TEAS, transcutaneous electrical acupuncture point stimulation.

**Table 3:** Data about the propofol dosage and procedure.

Variables	Control	TEAS	95% CI	*p* value
Induction dose [mg; median (P_25_, P_75_)]	124.0 (112.0, 136.0)	130.0 (112.0, 144.0)	−2 (−8, 2)	0.430
Maintenance dose [mg; median (P_25_, P_75_)]	52.0 (42.0, 61.0)	45.0 (26.0, 57.5)	6 (0, 12)	**0.011**
Total dose [mg; mean (SD)]	176.2 ± 26.0	172.0 ± 26.3	4.1 (−2.8, 11.1)	0.243
Procedure time [mins; median (P_25_, P_75_)]	15.7 (13.1, 16.9)	12.5 (10.4, 14.5)	2.3 (1.7, 3.2)	**< 0.001**
Awakening time [mins; median (P_25_, P_75_)]	4.5 (4.2, 5.2)	4.3 (3.3, 4.8)	0.5 (0.25, 0.73)	**< 0.001**

*Note:* Statistically significant results are in boldface.

Abbreviation: TEAS, transcutaneous electrical acupuncture point stimulation.

**Table 4:** Incidence of sedation-related adverse events.

Variables	Control	TEAS	*p* value
Hypotension [*n* (%)]	16 (14.7)	10 (9.2)	0.210
Bradycardia (< 50 beats min^−1^) [*n* (%)]	6 (5.5)	4 (3.7)	0.517
Emergence agitation [*n* (%)]	4 (3.7)	2 (1.8)	0.408
Cough [*n* (%)]	18 (16.5)	13 (11.9)	0.332
Hiccups [*n* (%)]	9 (8.3)	6 (5.5)	0.422
Nausea [*n* (%)]	8 (7.3)	5 (4.6)	0.391
Vomiting [*n* (%)]	7 (6.4)	4 (3.7)	0.353

*Note:* Bradycardia: < 50 beats/min.

Abbreviation: TEAS, transcutaneous electrical acupuncture point stimulation.

**Table 5:** Satisfaction of patients, anesthesiologists, and endoscopist.

Variables	Control	TEAS	*p* value
Patient satisfaction score [median (P_25_, P_75_)]	7.0 (6.0, 8.5)	8.0 (8.0, 9.0)	**< 0.001**
Anesthetist satisfaction score [median (P_25_, P_75_)]	8.0 (7.0, 8.5)	9.0 (8.0, 9.0)	**< 0.001**
Endoscopist satisfaction score [median (P_25_, P_75_)]	8.0 (6.0, 8.0)	9.0 (8.0, 9.0)	**< 0.001**

*Note:* Statistically significant results are in boldface.

Abbreviation: TEAS, transcutaneous electrical acupuncture point stimulation.

## Data Availability

The raw data supporting the conclusions of this article will be made available by the authors without undue reservation.
